# Effects of an Intensive 6-Week Rehabilitation Program with the HUBER Platform in the Treatment of Non-Specific Chronic Low Back Pain: A Pilot Study

**DOI:** 10.3390/clinpract12040064

**Published:** 2022-08-09

**Authors:** Mélanie Tantot, Vincent Le Moal, Éric Mévellec, Isabelle Nouy-Trollé, Emmanuelle Lemoine-Josse, Florent Besnier, Thibaut Guiraud

**Affiliations:** 1 Treboul Functional Rehabilitation Center, ORPEA/CLINEA, 29100 Douarnenez, France; 2 Research Center and Centre ÉPIC, Montreal Heart Institute, Montréal, QU H1T 1N6, Canada

**Keywords:** low back pain, rehabilitation, physical activity

## Abstract

Non-specific chronic low back pain (NSCLBP) is defined as a complex disorder involving structural, biomechanical, cognitive, psychological, social, and lifestyle factors. Non-pharmacological approaches such as exercise and physical therapy have been proposed in first-line treatments, along with psychological follow-up and pain medication if needed. Our objective was to evaluate the effectiveness of an intensive rehabilitation program with HUBER (a multi-axis motorized platform equipped with force sensors, allowing patients to perform physical exercises in an isometric mode) on the spine flexion-to-extension ratio at 60 and 120°/s, pain, and trunk flexibility in individuals with NSCLBP. Twelve participants underwent a clinical evaluation including isokinetic spine strength and participated in a 6-week rehabilitation program with HUBER 360 Evolution. The main findings of this pilot study show that the flexor/extensor ratios at 60°, the flexibility of the hamstring and quadriceps, and muscular endurance of the trunk, disability, and quality of life were significantly improved at the end of the rehabilitation program (*p* < 0.05). Low back pain and analgesic medication were also reduced. Exercising with the HUBER Platform seems to be effective in managing NSCLBP but a randomized study with a larger sample size and a control group is necessary.

## 1. Introduction

The prevalence of low back pain (LBP) increases with age and the prevalence of a sedentary lifestyle [1]. Among individuals experiencing LBP, some of them become chronic and disabled, labeled non-specific chronic low back pain (NSCLBP) [2]. The main etiologies of NSCLBP entail mechanical disorders, including injured intervertebral disc, injury to a facet joint or sacroiliac joint, osteoarthritis, and lumbar spinal stenosis [3]. Infectious, vascular, rheumatic diseases, and gynecological factors are non-mechanical factors also associated with NSCLBP [3,4]. Specifically related to the trunk, decreased muscle mass and strength in the trunk and the lumbar musculature have been associated with NSCLBP [5]. Trunk neuromuscular response is also decreased [6]. NSCLBP leads to reduced global muscle strength, endurance capacity and mobility, and reduced ability in activities of daily living [2,7]. Altogether, NSCLBP severely affects the quality of life of patients, resulting in disability and work absence [1,2].

NSCLBP is no longer considered an isolated musculoskeletal problem. This pathology is defined as a complex disorder involving structural, biomechanical, cognitive, psychological, social, and lifestyle factors [2,8]. The diagnosis of non-specific low back pain implies no known pathoanatomical cause [2] and diagnostic investigations have no role in the management of NSCLBP. Diagnostic tests only have a role when the clinician suspects a specific disease process that would be treated differently for non-specific low back pain [7].

According to guidelines, non-pharmacological approaches such as exercise and physical therapy have been proposed in first-line treatments along with psychological follow-up and pain medication if needed [2,8,9,10]. Physiotherapy, used in a comprehensive NSCLBP rehabilitation program, aims to decrease chronic pain and promotes proprioceptive and postural work, as well as spinal mobility, flexibility, and muscle-strengthening of the spinal area [2,9,10,11,12,13]. The effects of specific types of exercise treatments on pain intensity and functional limitation outcomes, and the most effective exercises in the management of NSCLBP, are still in debate [11]. Multidisciplinary and comprehensive rehabilitation programs also serve to reduce the patient’s anxiety, using reassurance strategies, recommendations to stay active and education. The objective of these programs is also to improve the quality of life, and alleviate fear–avoidance beliefs about physical activity and work that may affect and contribute to their low back pain and resulting disability [1,2].

**Figure 1:**
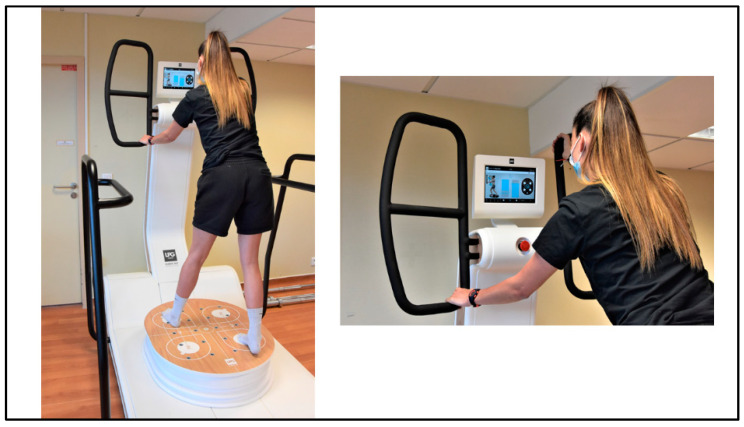
The HUBER 360 ^®^ Evolution Platform. Coordination and muscle-strengthening exercise on the HUBER Platform. The platform moves back and forth and left and right. The patient must push on the handles with the arms, but the movement starts from the legs with the core engaged. The target is represented by a blue gauge on the screen.

Within physiotherapy care, the device HUBER 360 ^®^ Evolution (LPG^®^ Systems, Valence, France) seems to meet the requirements of a functional rehabilitation program to improve mobility, strength, and pain in NSCLBP individuals. The HUBER 360 ^®^ Evolution is a multi-axis motorized platform equipped with force sensors, allowing patients to perform exercises that simultaneously elicit balance, coordination, and strength training in an isometric mode (Figure 1). The platform can move back and forth and side to side, and the handles mounted on a movable column are raised or lowered to permanently create an imbalance. The patient must maintain pressure on the handles. The target is represented by a blue gauge on the screen. The higher the target, the harder they have to push or to pull. The effect on an exercise training program with the HUBER Platform has already been studied in healthy older adults [14,15,16], in older women with mild cognitive impairment [17], and in individuals with cardiovascular diseases [18,19]. The published exercise programs with the HUBER Platform last from 4 to 8 weeks, with a number of sessions ranging from two to four sessions per week for a duration of 30 min each. The HUBER Platform safely improves body composition, balance, strength, cardiorespiratory fitness, and cognitive function in healthy older adults and cardiac patients [14,15,16,17,18,19]. To our knowledge, only one study has been conducted on individuals with NSCLBP using HUBER [20], but other types of platforms are under study [21]. In the study of Letafatkar et al. [20], significant improvements in proprioception, lumbar movement control, pain, and quality of life were observed after 5 weeks (10 sessions) compared to a control group. One of the limitations of this latest study is the lack of isokinetic trunk flexor/extensor muscle strength measurements. Isokinetic dynamometry examines the joint range of motion, trunk flexion, and extension strength at various angular velocities [22]. With an isokinetic exercise, the patient develops constant force throughout the movement with maximum muscle strength. The speed and the direction of the movement are constant regardless of the force applied by the subject. Isokinetic strength testing is a useful approach to assess trunk extension and flexion in healthy individuals as well as in patients with low back pain [22,23,24,25]. The trunk flexor and extensor muscle strength ratio are commonly tested to describe unilateral antagonist-to-agonist strength properties, functionality, and imbalances [22]. Isokinetic concentric strength assessment at 60 and 120°/s is one of the most commonly used criteria to analyze trunk flexor and extensor muscle strength [23,25]. Low-speed movements at 60°/s are more reliable for assessing muscle strength, whereas the flexors/extensors ratio at 120°/s most closely approximates muscle balance in the activities of daily living of patients [24,25].

The objective of this pilot study was therefore to evaluate the effectiveness of an intensive care program with HUBER 360 ^®^ Evolution on the spine flexion-to-extension ratio at 60 and 120°/s, trunk flexibility, pain, and disability in individuals with NSCLBP.

## 2. Materials and Methods

### 2.1. Patients

This observational study was conducted at the Rehabilitation Center of Tréboul (Douarnenez, France) between April and September 2021 and involved 12 participants. The clinical program was on a voluntary basis and a medical prescription because of chronic low back pain.

The clinical evaluation included the search for warning signs (such as neurological symptoms, paresthesia, significant trauma, significant structural deformity of the spine, etc.). These “red flags” point to an underlying pathology requiring specific and/or urgent treatment. The search for chronicization factors (such as inappropriate pain behaviors, especially avoidance of or reduction in activity, related to fear, work-related issues, etc.) was also undertaken. The clinical examination included an evaluation of spinal mobility, the flexibility of the muscles of the lumbo–pelvic–femoral complex, and the muscular endurance of the muscles of the trunks and lower limbs. A peripheral neurological assessment was also carried out in the event of irradiation in the lower limbs. A precise questioning of the patient’s pain, his experience in relation to his pathology, the history of his low back pain, and his beliefs was also carried out.

Eligibility criteria for the rehabilitation program were: age between 20 and 60 years old with NSCLBP (more than 3 months), with clinical and radiological assessment. The exclusion criteria were chronic low back pain of specific etiology (trauma, tumor, inflammatory or infectious disease, or radicular syndrome), spine with major anatomical deformations, any contraindication for the rehabilitation program, surgery less than 3 months, and/or receiving treatment with corticosteroids. All subjects gave their informed consent for inclusion before they participated in the study.

### 2.2. Measurements

All participants underwent a complete clinical evaluation including isokinetic spine strength with a CON-TREX (TP-500, Physiomed, Schnaittach, Germany). The spinal flexors and extensors were explored at speeds of 60 and 120°/s, in line with the guidelines [22]. After a 10 min warm-up period on a cycle ergometer, patients were set up in a standing position. Their legs, pelvis, and chest were kept in place with fastening material. The protocol started with 10 continuous passive mobilizations at 15°/s. After 1 min of recovery, 6 consecutive submaximal bending-extension movements with trunk ante-flexion at 60°/s were performed. After another 1 min recovery, a maximal evaluation was performed at 60°/s with 3 repetitions at an amplitude of −10–60°. The same protocol was applied at 120°/s. To avoid inter-operator variability, the same operator performed all tests for a specific patient (See the measurements section of the Supplemental Digital Content for details).

The following examinations were also performed at baseline and at 6 weeks: 1/flexibility of the lower limbs (the hamstring, psoas, and quadriceps), 2/spine joint mobility (double-inclinometer method), 3/muscular endurance of the trunk (Shirado–Ito and Sorensen tests) and the lower limbs (Killy test), 4/pain (visual analogue pain scale graduated from 0 to 10), 5/questionnaires (Fear and Avoidance Belief Questionnaire (FABQ [26]) and Oswestry Disability Index [27]), 6/cardiorespiratory fitness (submaximal test to predict VO2max [28]), and 7/analgesic prescription. (See the measurements section of the Supplemental Digital Content for details).

### 2.3. Rehabilitation Program

All patients benefited from standard care. The rehabilitation program included 24 sessions, spread over 6 weeks with 4 sessions of 2 h per week supervised by a physiotherapist. Each 2 h session included (each time in the same order) 1 h of rehabilitation, 30 min of balneotherapy, and 30 min of HUBER 360 (Figure 1), all with mobility, flexibility, and muscle-strengthening exercises. Specifically, these exercises included self-awareness of the lumbo–pelvic–femoral complex and multidirectional mobility of the lumbar spine. We gradually integrated aerobic exercises and muscle strengthening of the lower limbs, trunk, and spine extensors with bodyweight exercises. HUBER exercises require the synergistic activation of various muscle groups of the lower limbs, trunk, and upper limbs to develop low–high force levels against the handles. The exercises included pulling and pushing exercises on the handles. The placement of the feet on the platform and the hands on the handles varied according to the exercises proposed. Each HUBER session included 3 types of exercises: mobility, stretching, and muscle strengthening. For mobility exercise, this mainly concerned the lower limbs, the pelvis, and the lumbar and thoracic spine. Mobilization times were 10 to 15 s, with varied amplitudes adapted to the patients, followed by 10 s of active recovery (where the amplitude is less important), for 8 to 12 sets per region mobilized. Stretching exercises (20 s followed by 10 s recovery) mainly targeted the posterior chain (lower limbs and thoracic and lumbar spine). Patients performed 10–12 repetitions for each muscle group. Muscular strengthening (spine, lower, and upper limbs): the first 2 weeks mainly included isometric strengthening (quadriceps, triceps surae, hamstrings, glutes...). The force level ranged from 40 to 50% of the maximum voluntary contraction during the first 2 weeks and then increased progressively according to the participant. Participants performed between 10 and 15 isometric contractions per exercise, ranging from 30 to 45 s with 10 to 15 s of passive recovery at the beginning of the program. The handles were equipped with strain gauges, and feedback about the force developed was provided to users. An interactive interface, shown as a target, informed the subject about their ability to maintain the required force level. (See the rehabilitation exercises section of the Supplemental Digital Content for details.) 

### 2.4. Research Plan/Design

Following the medical evaluation during the first day, the research’s procedures were explained to patients. Once the consent form has been signed, all evaluations were carried out within 2 days. Then, the participants began the rehabilitation program 4 times per week for 6 weeks. Post-rehabilitation evaluations took place during the last 2 days of the stay under the same conditions as at baseline and in the same order.

### 2.5. Statistical Analysis

All data are expressed as mean ± standard deviation (SD), or in number and percentage. Histograms and Shapiro–Wilk tests were used to control the normality of the distribution for each variable. When the assumptions were met, a paired Student’s *t*-test was used to compare the effectiveness of the intervention (based on the change between post-intervention and pre-intervention data). Otherwise, Wilcoxon signed-rank test (for non-normal distribution) was used. The effect size (Cohen’s d) was computed to evaluate the strength of the intervention, by using the mean of the delta (post–pre) divided by the mean of the standard deviation of the delta. Univariate and multivariate regressions were used to explore the potential predictors of trunk muscle strength (isokinetic trunk flexion/extension ratio torque at 60 and 120°/s) and pain. Potential predictors with *p* values < 0.20 in the univariate model were included in a stepwise multivariate regression analysis. All statistical tests were two-sided and conducted at a 0.05 significance level. Statistical analyses were performed with the use of Stata SE 15.1 (StataCorp LP, College Station, TX, USA).

## 3. Results

Twelve individuals with NSCLBP completed all the examinations. The baseline and post-intervention characteristics are reported in Table 1. Briefly, after the intervention, significant improvement was observed in isokinetic trunk flexion/extension ratio torque at 60°, peak torque at 60° and 120°, pain score and analgesic treatment, the flexibility of the hamstring and the quadriceps, muscular endurance of the trunk and the lower limbs, and cardiorespiratory fitness (all *p* < 0.05), with large effect size (Cohen’s d ranging from 0.68 to 1.63). The Oswestry Disability Index and the Fear and Avoidance Belief Questionnaire scores were also significantly improved (*p* < 0.001 and *p* < 0.05, respectively).

Lumbar ranges of motion in flexion, extension, right and left lateral flexion, as well as the flexibility of the hip flexors, were not statistically different.

**Figure 2:**
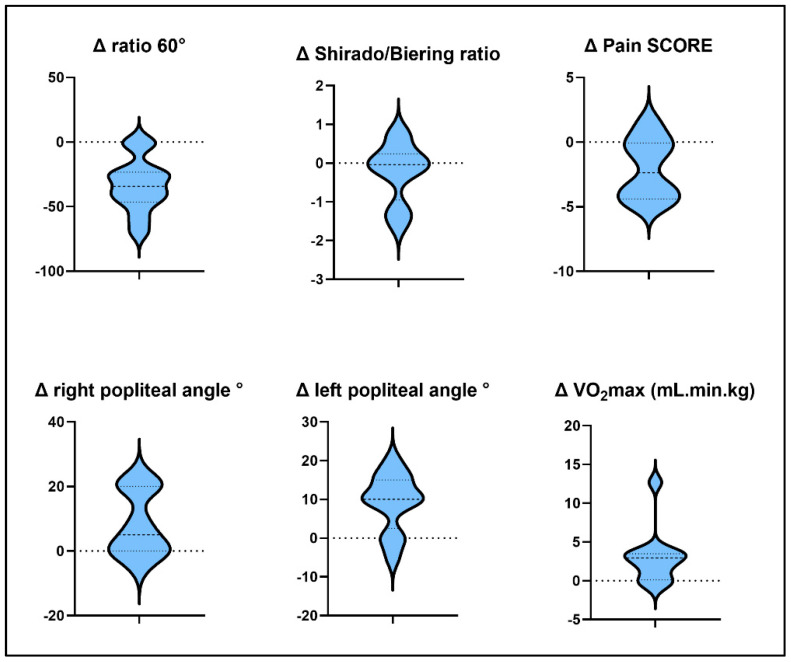
Violin box representing change between pre and post intervention data. The black dotted lines represent the median and the interquartile range.

The individual changes in the main outcomes are presented in Figure 2 (violin box). A decrease in pain score was negatively correlated with the flexibility of the hip flexors on the right side only (*p* = 0.023), such that improvement on the modified Thomas test was associated with lower pain scores.

No independent predictors (stepwise multivariate model) for Δ isokinetic trunk flexion/extension ratio torque at 60° or Δ pain score were found.

## 4. Discussion

The main findings of this study show that the flexor/extensor ratios and peak torque at 60°, the flexibility of the hamstring and quadriceps, muscular endurance of the trunk, disability, and quality of life were significantly improved at the end of the rehabilitation program. Low back pain and analgesic medication were also reduced. The large effect sizes indicate that our findings are clinically relevant to the main outcomes.

The HUBER platform is an alternative form of exercise known to have a positive effect on body composition, balance, strength, cardiorespiratory fitness, and cognitive function in different populations [15,16,17,18,20,29]. In NSCLBP, Letafatkar et al. reported significant improvements in the proprioceptive system, movement control, and quality of life in the HUBER group compared to a control group [20]. In our pilot study, the exercises on the HUBER oscillating platform were carried out with the platform moving from front to back but also from left to right, in order to create a permanent imbalance to stimulate the stability of the person. The proprioceptive receptors are placed at the foot level, and in the pelvic region, and the muscles of the trunk are stimulated to maintain this balance. At the same time, the person must push or pull on the handles depending on the exercise. Rehabilitation exercises on mobile or even unstable surfaces effectively stimulate the proprioceptive system and provide permanent feedback to maintain balance and spatial awareness [20,21]. Our findings are in line with those of Letafatkar et al. [20], adding significant improvements in isokinetic spine strength, lumbar joint mobility, flexibility, and muscular endurance of the trunk and the lower limbs. The rotating platform’s range of motion variations stimulate the lumbar muscles and the deep abdominals. This allows strength development at both superficial and deep muscle levels, essential to maintaining good postural balance, coordination, and proprioception. Our program is also effective in improving the endurance time of the core muscles, which could lead to reduced low back pain symptoms and recurrence [29,30].

Lumbar stabilization exercises seem to improve isokinetic data such as the F/E ratio at 60°/s, quality of life, and pain scores. In the study by Melo et al., 2014, the F/E ratio at 60°/s was changed from 169.1% to 107.4% after rehabilitation [31]. In our study, the F/E ratio at 60°/s changed from 104.7% (±25.03) to 71.25% (±14.72). A normal flexor/extensor ratio should be between 0.6 and 0.7 [25]. In another study, the F/E ratio at 120°/s was significantly improved from 112% (±30) to 107% (±23) after daily exercise training with an isokinetic protocol [32]. In our study, this ratio at 120°/s changed from 89.78% (±23.68) to 78.25% (±11.27). The strength of the trunk muscles of the subjects was significantly improved by our protocol with HUBER, while a recent meta-analysis showed that there is no superiority of resistance exercises or stabilization exercises compared to control groups on core muscle strength in NSCLBP [33] (the standardized mean difference between the stabilization exercises and the control groups was 0.21 (95% CI −0.28 to 0.69), *p* = 0.399). In chronic low back pain patients, there is a strength imbalance in favor of a lack of spinal extensor strength, and the ratio is therefore generally greater than 1 in these patients [22,25]. Lower extensor muscle strength than flexor muscle strength might be one risk factor for low back pain [34]. Indeed, flexor/extensor ratio imbalances have been related to back pain [35,36]. The severity of low back pain is associated with decreased isometric and isokinetic strength of trunk muscles [5]. In our study, the lack of subjects does not allow us to find a relationship between the improvement in the F/E ratio and the reduction in pain.

The Oswestry Disability Index and the Fear and Avoidance Belief Questionnaire scores were significantly improved in our study, suggesting reduced disability and improved quality of life. In the systematic review and meta-analysis evaluating the efficacy of kinesio taping vs. other general physical therapies, kinesio taping had a more positive effect on improving pain intensity and Oswestry Disability Index than other methods of conventional therapy [37]. The weighted mean difference between kinesio taping vs. conventional therapy for the Oswestry Disability Index was –7.11 (95% CI –8.70 to –5.51) in favor of kinesio taping [37]. The global mean change for the Oswestry Disability Index for conventional therapy (grouping together eight interventional studies with physical therapy) was −11.4 after the rehabilitation vs. −19.1 for kinesio taping [37]. In our study, the Oswestry Disability Index score change was −15.8 ± 2.2 (95% CI −20.7 to −11.00) after the 6-week program, suggesting that exercising on the HUBER platform is effective in reducing functional limitation. Comparative studies are necessary between the different management techniques. 

Management of chronic low back pain currently comprises a range of intervention strategies, such as supervised exercise therapy and manual therapy. There is still debate with regard to the most appropriate form of exercise and there is no evidence to support the superiority of one form of exercise over another in the treatment of NSCLBP [12]. McKenzie therapy and Pilates seem to be more effective than other types of exercise treatment for reducing pain intensity and functional limitations [11]. However, in two Cochrane reviews studying the effects of Pilates [38] (10 trials; *n* = 510) and Motor Control Exercise [39] (32 trials; *n* = 2628), and in one systematic review and meta-analysis comparing stabilization exercises vs. manual therapy [40] (11 trials; *n* = 895), the authors concluded that there was no clinical evidence that one method was superior to other forms of exercises. The decision to use one method of rehabilitation rather than another for chronic low back pain may be based on the patient’s or care provider’s preferences. Further studies are needed to compare the programs with each other. Nevertheless, some studies have found that exercise intensity plays an important role in improving disability and exercise capacity in NSCLBP [13,41]. Altogether, long-term, consistent, individualized, exercise-based treatment approaches are most likely to result in improvements in pain and function [42].

Previously, Markovic et al. [16] compared the effects of an 8-week program (three times per week) with the HUBER platform vs. Pilates training on balance ability, neuromuscular function, and body composition in healthy older women [16]. Although the Pilates sessions lasted 1 h compared to 30 min for the sessions with HUBER, the authors showed that balance and core resistance training with the HUBER platform were more efficacious in improving balance ability, trunk muscle strength, leg power, and body composition when compared to traditional Pilates training [16]. The HUBER platform is an ‘‘all-in-one’’ device, associating balance, coordination, cognitive, and strength training, and may have a place in therapy for NSCLBP. Furthermore, the exercise program was guided through an application (available on the screen), which required less supervision by the physiotherapists once the patient was independent and understood the exercises.

Some limitations of our pilot study should be mentioned: the absence of a control group without a physical activity program or without exercise with the HUBER platform does not allow the results to be generalized. A randomized study with a larger sample size with an exercising control group without the HUBER platform is necessary in order to compare the effectiveness of the use of this equipment. Furthermore, the medium–long-term effects were not explored in this study (i.e., at 6 months). A follow-up visit at 6 months would allow us to study whether patients maintain healthy lifestyle habits with regular physical activity and good spinal mobility without pain. Finally, men and women can exhibit different pain sensitivities [43]. Our pilot study does not allow a sex-difference evaluation but this aspect should be considered in future studies.

Altogether, our results are promising. Exercising with the HUBER Platform seems to be effective in managing NSCLBP in most of the clinical parameters within 6 weeks only. A randomized study with a larger sample size and a control group is necessary. A longer-term follow-up would also allow the persistence of benefits to be studied.

## Figures and Tables

**Table 1:** Clinical Characteristics at Baseline and After the 6-week Intervention Program.

	HUB *n* = 12		*p*	Cohen’s d
	Pre	Post		
Age	42.92 ± 9.17			
Sex; *n* female (%)	3 (25%)			
Weight (kg)	89.00 ± 23.78			
Heigh (cm)	175.33 ± 6.85			
Body mass index (kg·m^2^)	29.08 ± 8.07			
Pain visual analog scale (/10)	3.70 ± 1.92	1.65 ± 1.28	0.0110	1.26
Oswestry Disability Index	29.83 ± 9.32	14.00 ± 8.36	0.0000	1.79
FABQ * work	73.11 ± 18.58	50.63 ± 32.04	0.0244	0.86
FABQ * physical activity	54.12 ± 23.06	27.43 ± 15.74	0.0275	1.35
Iso Fr 60 (Nm)	197.77 ± 99.81	209.17 ± 64.46	0.1823	0.14
Iso Exr 60 (Nm)	209.03 ± 114.12	301.33 ± 99.85	0.0000	0.86
Ratio 60	104.70 ± 25.03	71.25 ± 14.72	0.0002	1.63
Peak Torque moy./kg 60 (Nm/kg)	2.12 ± 0.95	3.27 ± 0.92	0.0000	1.24
Iso Fr 120	177.83 ± 80.90	212.43 ± 82.52	0.0006	0.42
Iso Exr 120	210.76 ± 112.48	267.32 ± 76.60	0.0149	0.59
Ratio 120	89.78 ± 23.68	78.25 ± 11.27	0.1487	0.62
Peak Torque moy./kg 120 (Nm/kg)	2.32 ± 0.98	3.01 ± 0.77	0.0090	0.78
VO_2_max (mL. kg. min)	31.80 ± 5.00	34.80 ± 5.23	0.0076	0.59
Lumbar mobility and flexibility	Pre	Post	*p*	Cohen’s d
Dual Inclinometry Flex° (°)	23.33 ± 8.88	19.17 ± 5.57	0.0854	-
Dual Inclinometry Ext° (°)	18.25 ± 12.59	23.33 ± 13.54	0.147	-
DI Inclination Right (°)	14.17 ± 7.93	17.92 ± 6.56	0.095	-
DI Inclination Left (°)	14.08 ± 8.32	17.92 ± 5.82	0.1499	-
Modified Thomas test Right (°)	18.06 ± 10.58	13.75 ± 9.32	0.1485	-
Modified Thomas test Left (°)	13.89 ± 7.73	13.75 ± 11.10	0.9647	-
Popliteal angle test Right (°)	138.06 ± 11.99	146.25 ± 9.56	0.0166	0.76
Popliteal angle test Left (°)	136.53 ± 12.28	145.83 ± 8.75	0.0012	0.87
Distance heel–buttocks Right (cm)	10.67 ± 6.95	4.42 ± 4.03	0.0012	1.10
Distance heel–buttocks Left (cm)	9.67 ± 7.88	5.58 ± 4.91	0.0278	0.62
Biering–Sorensen test (s)	67.67 ± 38.49	113.83 ± 56.33	0.0102	0.96
Shirado–Ito test (s)	66.67 ± 37.00	106.33 ± 59.71	0.0051	0.80
Shirado/Biering ratio	1.28 ± 1.12	1.05 ± 0.64	0.3228	
Killy test (s)	38.50 ± 22.89	225.92 ± 229.21	0.0022	1.15
Pharmacological treatments (number of patients concerned with each class of therapy)				
Analgesic step 1/2/3 *	6/3/3	4/1/1		
Antidepressant	2	2		
Antiepileptic	1	1		
ACE * inhibitors	1	0		

* ACE: Angiotensin-converting enzyme (ACE) inhibitors; FABQ: Fear and Avoidance Belief Questionnaire. Analgesic steps from 1 to 3 refer to the WHO Pain Ladder with Pain Management Guidelines (step 1: Mild Pain (1–3/10); step 2: Moderate Pain (4–6/10); step 3: Severe Pain (7–10/10).

## Data Availability

The data of our pilot study are available for sharing purposes from the principal investigator under reasonable request.

## Supplementary Materials

The following supporting information can be downloaded at: https://www.mdpi.com/article/10.3390/clinpract12040064/s1, Materials and Methods S1: Measurements.
