# Supplementary material for: Effects of an Intensive 6-Week Rehabilitation Program with the HUBER Platform in the Treatment of Non-Specific Chronic Low Back Pain: A Pilot Study
Source: Clin Pract. 2022 Aug 9;12(4):609–18. doi: 10.3390/clinpract12040064 (PMC9406810; doi:10.3390/clinpract12040064)
Supplement: Supplementary file 1 [file clinpract-12-00064-s001.zip › clinpract-1848819-supplementary.pdf]

Supplementary Text

Measurements

1/ Isokinetic spine strength (CON-TREX, TP-500, Physiomed, Germany)

The spinal flexors and extensors were explored at speeds of 60 and 120˚/s. Patients first performed a 10-minute warm-up on a cycle ergometer before the isokinetic procedure. Patients were then set up in a standing position, and their legs, pelvis and chest are kept in place with fastening material. A preliminary phase was carried out with less than maximal exertion. The patient was informed of the apparatus's operational principle and was given advice on how to produce maximum repetitive effort throughout their movements, whether they consist in bending or in extending. The protocol started with 10 continuous passive mobilization at 15°/s. After 1 min of recovery, 6 consecutive submaximal bending-extension movements with trunk ante-flexion at 60°/s were performed. After another 1 min recovery, a maximal evaluation was performed at 60°/s with 3 repetitions at an amplitude of 10–60˚. The same protocol was applied at 120°/s. The maximum moment of force (MMF) in Newton-meters (Nm) for the 2 speeds during the 3 repetitions recorded; it is the moment of highest force during a series. The mean power (MP) in watts (W), the total workload (TW) in Joules (J) were recorded at both 60 and 120°/s. The flexor/extensor ratios at 60 and 120°/s are the flexor/extensor ratios for the aforementioned parameters.

2/ Flexibility of the trunk

Flexibility testing of the hamstring, psoas and quadriceps muscles were performed.

Hamstring: The patient was lying in the supine position with the contralateral lower limb extended on the table. The therapist then flexed the hip at 90°, brought the knee to an extension until their limit (according to the sensations of the patient). An inclinometer was placed on the anterior tibial tuberosity to measure the angle (in degrees) of the popliteal between the tibia and the femur on both legs.

Psoas: the modified Thomas' test was used to measure the flexibility of the hip flexors, which included the iliopsoas muscle group. The patient laid on their back, with both legs hanging freely at the edge of the table. First, they achieved a maximum flexion of both knees using both arms to ensure that the lumbar spine is flexed and flat on the table to avoid an anterior tilt of the pelvis.

The patient then lowered the tested limb toward the table, whilst the contralateral hip and knee were held in maximal flexion to stabilize the pelvis and flatten out the lumbar lordosis. Once the final position was reached, the inclinometer was placed along the midline of the femur, between the greater trochanter and the lateral femoral condyles (in degrees). The length of the iliopsoas was measured by the angle of the hip flexion.

Quadriceps: Ely's test heel-buttock distance was used to measure the flexibility of the quadriceps. The patient laid in the prone position. The therapist stood next to the patient, at the side of the leg that is tested. The patient's knee was flexed to bring their heel as close as possible to their buttock. One hand was on the lower back, the other holding the leg at the heel. The closest distance between the heel and the buttock (in cm) was measured. The test is done on both sides for comparison.

3/ Lumbar joint mobility

The Dual inclinometer technique was used to assess the spinal lumbar range of motion in flexion, extension, right and left lateral flexion.

Flexion/Extension: The participant was placed in a neutral position. The inclinometers were set to 0 and placed on the thoracolumbar junction (T12-L1) and sacrum (S1). The patient leaned forward as far as possible, with the arms left in front and without bending the knees. Then the value of the 2 inclinometers was registered. The degree of lumbar flexion is the subtraction of the inclinometer figure of S1 from that of T12-L1 (in degrees). The same process is used for lumbar extension when the patient leans back as far as possible with his arms at his sides. S1 was subtracted from T12-L1.

Lateral Flexion: The inclinometers were placed on the frontal planes of both the S1 and T12 vertebrae. The participants were asked to laterally flex to the right by running their right hands down the lateral thigh towards the right knee. The readings were then taken from the two inclinometers. The difference between the T12 and the S1 inclinometers gave the true right lateral flexion value. The right lateral flexion procedure was repeated for left lateral flexion.

4/ muscular endurance of the trunk

Shirado-ito test: is a static endurance test of the abdominal muscles currently used in the assessment of low back pain. The participant laid on their back with their hips and knees bent at 90 °, the heels were placed on a box. The arms crossed on the chest and the hands on the shoulders.

The shoulder blades were off the ground, the neck flexed. The test was stopped if the shoulder blades were lowered or if the patient was exhausted. The time to hold the position is timed.

Sorensen test: consisted of an isometric contraction of trunk extensor muscles. The participant laid in a prone position, on a roman chair, with the iliac spines fixed on the edge of the support. The lower limb was blocked by a module placed on the back of the leg, above the heel. The chest was perfectly straight, neither too arched nor too bent. The objective was to maintain the position as long as possible, as soon as the torso drops the timer is stopped.

5/ muscular endurance of the lower limbs

Killy test for evaluating isometric knee extensor endurance was performed. The participant pressed their back against a wall. Hips, knees, and ankles are flexed to 90°. Arms along the body. The test was timed.

6/ pain intensity evaluation

A visual analogue pain scale graded from 0 to 10 was used to quantify the amount of pain that a patient feels from none (0) to an extreme amount of pain (10).

7/ questionnaires

Fear and Avoidance Belief Questionnaire (FABQ) [1]: is a self-reported questionnaire which specifically focuses on how a patient's fear avoidance beliefs about physical activity and work may affect and contribute to their low back pain and resulting disability. Sixteen questions scaled from 0 to 6 (a higher score indicates fear avoidance behaviors). The Physical Activity subscale (FABQ-PA) ranges from 0 to 24 and the Work subscale (FABQ-W) ranges from 0 to 42.

Oswestry Disability Index [2]: is a self-completed questionnaire containing ten topics concerning the intensity of pain, lifting, ability to care for oneself, ability to walk, ability to sit, sexual function, ability to stand, social life, sleep quality, and ability to travel. Each topic category was followed by 6 statements describing different potential scenarios in the patient's life relating to the topic. The patient checked the statement which most closely resembled their situation. Each question was scored on a scale of 0–5 with the first statement being zero and indicating the least amount of disability and the last statement is scored 5 indicating the most severe disability. The scores for all

questions answered are summed, then multiplied by two to obtain the index (range 0 to 100). Zero is equated with no disability and 100 is the maximum disability possible.

8/ cardiorespiratory fitness

To predict VO2max, the Canadian Aerobic Fitness Test was performed [3]. The test is submaximal. Subjects step up and down a double step (40.6 cm height), following the instructions and stepping rhythm as the determined metronome, based on their age and sex. Stepping was performed with a six-pace cycle: one foot on the middle step, two on the top step, one on the middle step, and both feet on the ground. The subject started stepping for 3 minutes at a rhythm appropriate to the individual's age. Pulse rate was measured for 10 seconds (between 5 & 15 seconds after stepping). If the pulse rate was within a specified safety zone (see table of halting criteria), stepping was recommenced at 3 minutes 25 seconds. If the pulse ceiling still had not been reached, the subject continued for a third stage, at a stepping rate appropriate to a person who was 10 years younger than themselves. It is possible to convert the test result to a predicted VO2max score, using the equation by Jette et al. [3]: *VO2max (mL/kg/min) = 42.5 + 16.6 (E) - 0.12 (M) - 0.12 (HR) - 0.24 (A).* Where E is the energy cost of the final test stage in L/min (see table in [3]), M is the body mass in kg, HR is the heart rate in beats/min and A is the subject's age in years.

9/ Rehabilitation exercises

<u>The first two weeks</u> mainly consist of lumbar and pelvic joint mobility exercises with a physiotherapist. These exercises help reintegrate these underused areas into the patient's motor pattern. Learning of all the functional movements used during the program is also carried out (squats, lunges, empty deadlifts, work of the transverse muscle...). Stretching exercises are also performed. The balneotherapy program uses the same main principles but with higher joint and muscle amplitudes (depending on the patient's tolerance) given the reduction of gravity in the water. On the HUBER platform, patients performed low levels of balance exercises, spinal and hip mobility, and coordination. The exercises included pulling and pushing exercises on the handles. The placement of the feet on the platform and the hands on the handles varied according to the exercises proposed. Each session lasted 30 minutes. The force level ranged from 40 to 50% of the maximum voluntary contraction during the first 2 weeks. Participants performed between 10 and 15 isometric contractions per exercise ranged from 30 to 45 seconds with 10-15 seconds of passive recovery. An interactive interface with bar graph materialized as a target, informed the subject

about their ability to maintain the required force level. Gentle muscle stretching was initiated, especially in the lower limbs (triceps surae, hip abductors, quadriceps, hamstrings). A physiotherapist or a kinesiologist supervised all the exercises sessions.

Over the next two weeks, more intense muscle strengthening exercises were integrated. All the exercise sessions were supervised by a physiotherapist or a kinesiologist. Exercises became more complex, recovery times decreased and work times increased. Aerobic exercises such as interval-training, climbing stairs, circuit training, etc.) were also performed. The strengthening of the spinal extensor muscles started with body weight only and then followed by weight training (deadlifts, squats and kettlebell swings). In balneotherapy, the exercises are more focused on muscle strengthening of the lower limbs and the trunk as well as on the cardiorespiratory capacities of the patients. Jumps are also initiated during this phase. Mobility programs were maintained on the HUBER but their intensities were increased to reach a moderate / slightly difficult intensity. Muscle strengthening and high-intensity interval training sessions focused on the lower limbs, core and upper body. Each isometric contraction ranged from 30 to 45 seconds followed by 10 seconds of passive recovery with 20 and 30 contractions per exercise.

Finally, during the last two weeks patients maintained what they have learned in terms of exercises (how to warm up, what exercises to do……etc…). In balneotherapy as in physiotherapy, the intensity of the exercises is increased to reach a fairly difficult intensity for interval exercise training and circuit training. On the HUBER platform, more complex exercises in terms of coordination and balance (with different placement of feet on the platform and hands on the handles), and muscle-strengthening were added to the mobility and high-intensity interval exercises. The platform moved back and forth and side to side, and the handles were raised or lowered by raising or lowering the movable column during isometric exercises to permanently create an imbalance. The patient had to maintain pressure on the handles. The target was represented by a blue gauge on the screen. The higher the target, the harder they had to push or pull. Each isometric contraction ranged from 30 to 45 seconds followed by 10 seconds of passive recovery.
